# T cells cooperate with palmitic acid in induction of beta cell apoptosis

**DOI:** 10.1186/1471-2172-10-29

**Published:** 2009-05-22

**Authors:** Tamara Cvjetićanin, Ivana Stojanović, Gordana Timotijević, Stanislava Stošić-Grujičić, Djordje Miljković

**Affiliations:** 1 Department of Immunology, Institute for Biological Research "Siniša Stanković", University of Belgrade, Belgrade, Serbia; 2 Institute of Molecular Genetics and Genetic Engineering, University of Belgrade, Belgrade, Serbia

## Abstract

**Background:**

Diabetes is characterized by progressive failure of insulin producing beta cells. It is well known that both saturated fatty acids and various products of immune cells can contribute to the reduction of beta cell viability and functionality during diabetes pathogenesis. However, their joint action on beta cells has not been investigated, so far. Therefore, we explored the possibility that leukocytes and saturated fatty acids cooperate in beta cell destruction.

**Results:**

Rat pancreatic islets or insulinoma cells (RIN) were co-cultivated with concanavalin A (ConA)-stimulated rat lymph node cells (LNC), or they were treated with cell-free supernatants (Sn) obtained from ConA-stimulated spleen cells or from activated CD3^+ ^cells, in the absence or presence of palmitic acid (PA). ConA-stimulated LNC or Sn and PA cooperated in inducing caspase-3-dependent RIN cell apoptosis. The observed effect of PA and Sn on RIN cell viability was mediated by p38 mitogen-activated protein kinase (MAPK)-signaling and was achieved through auto-destructive nitric oxide (NO) production. The cooperative effect of Sn was mimicked with the combination of interleukin-1β, interleukin-2, interleukin-6, interleukin-17, interferon-γ and tumor necrosis factor-α.

**Conclusion:**

These results imply that stimulated T cells produce cytokines that cooperate with saturated free fatty acids in beta cell destruction during diabetes pathogenesis.

## Background

Diabetes mellitus is a common name for a group of metabolic diseases characterized by hyperglycemia resulting from defects in insulin secretion and/or action. Hyperglycemia and other related metabolic disturbances can lead to serious damage to various systems of the body, especially nerves and blood vessels. Statistical data show that the excess global mortality attributable to diabetes in the year 2000 was 5.2% of all deaths [1], while its prevalence for all age-groups worldwide was expected to increase from 2.8% in 2000 to 4.4% in 2030 [2]. The most frequent forms of diabetes mellitus are type 2 diabetes (T2D) and type 1 diabetes (T1D).

T2D is classically considered to be a metabolic disease characterized by insulin resistance and additional disorders, such as pancreatic beta cell dysfunction and decrease in beta cell mass, which intensively contribute to the disease course [3,4]. One major reason for insulin resistance in T2D is obesity [4,5], which is associated with high glucose and free fatty acid levels circulating throughout the body affecting various cell types including pancreatic beta cells [6]. It is well known that hyperlipidemia contributes to T2D pathogenesis [4,5,7], and that high concentrations of saturated fatty acids, including palmitic acid (PA), lead to impairment of insulin action and beta-cell dysfunction [6,8,9]. Increased adiposity enhances adipose tissue secretion of cytokines, such as TNF-α, IL-1β and IL-6 which are cytotoxic to beta cells, as well as production of adipokines, including leptin, resistin, and adiponectin, which have a cytoprotective role in cytokine and free fatty acid-induced beta cell destruction [10-12]. Obesity and insulin resistance have been frequently associated with a state of low-grade inflammation and therefore it is assumed that inflammation contributes in a major way to the development of T2D [4,13,14]. An accumulating set of data supporting this assumption includes findings of inflammation-related changes, such as amyloid deposits and fibrosis, increased beta cell death and the presence of leukocyte infiltrates in the pancreata of T2D patients and animals [4,15,16]. Additionally, it is becoming increasingly clear that obesity is also an important predisposing factor for T1D [4,5], a disease with proposed autoimmune etiopathology, in which immune cells infiltrate the endocrine pancreas and cause beta cell destruction [17]. The means by which macrophages and T cells exert their cytotoxic actions upon beta cells are rather well established and comprise various soluble mediators, such as oxygen free radicals, nitric oxide (NO) and cytokines, including IL-1β, IL-6, IFN-γ and TNF-α, but the primal target of the autoimmune attack has not yet been defined [17]. It has been suggested that physiological beta cell turnover may expose specific tissue antigens that are carried to the draining lymph nodes by infiltrating macrophages [17]. Importantly, this initial turnover may be a consequence of obesity, i.e. hyperlipidemia or the excess production of cytokines in adipose tissues [4].

Thus, in the present paper we investigated the interaction between one of the most common free fatty acid – PA and soluble immune cell products, as they have individually been shown to be deleterious for beta cells [8,11,18], but their joint effect on beta cells has not been investigated so far. Here, we demonstrate that PA and soluble products of T cells cooperate in beta cell destruction *in vitro*. We show that they trigger caspase-3-mediated, NO-dependent apoptosis in rat insulinoma cells through activation of p38 mitogen-activated protein-kinase (MAPK).

## Methods

### Reagents, cells and cell cultures

All reagents were from Sigma (St Louis, MO, USA) and all dishes for culturing cells from Sarstedt (Numbrecht, Germany), unless stated differently. To make the stock solution for further dilution in RPMI 1640, palmitic acid (PA) was mixed overnight at 37°C in Krebs Ringer HEPES buffer containing 20% BSA (fraction V, Roche, Basel, Switzerland). The following recombinant cytokines were used in the experiments: rat IFN-γ (R&D Systems, MI, USA, 10 ng/ml), mouse IL-17 (R&D Systems 50 ng/ml), mouse IL-1β (10 ng/ml), rat TNF-α (10 ng/ml), human IL-2 (100 ng/ml), rat IL-6 (R&D Systems, 10 ng/ml). SB202190 was used as a selective inhibitor of p38 MAPK activation and was applied to cell cultures at least 30 minutes prior to additional treatments. Hemoglobin (Hb, 20 mg/ml) was used as the NO quencher, and was applied to cultures at least 1 hour before additional treatments. Rat insulinoma cells RINm5F (RIN) were grown under standard conditions (37°C, 5% CO_2_) in RPMI 1640 medium supplemented with 5% fetal calf serum (FCS, PAA Chemicals, Pasching, Austria), L-glutamine, 2-mercaptoethanol and antibiotics (culture medium) in tissue culture flasks until reaching approximately 80% confluence. Then, they were detached with trypsin solution (0.25%) and ethylenediaminetetraacetic acid (EDTA, 0.02%) in PBS. Cells were washed and seeded into 96-well flat-bottom plates (1 × 10^4^/well) for the MTT test, cell-based ELISA and Griess reaction, into 24-well plates (1 × 10^5^/well) for co-cultivations, into 6-well plates (2.5 × 10^5^/well) for cytofluorimetric analysis, or tissue culture flasks (25 cm^3^, 1 × 10^6^) for western blot, ca. 16 hours before treatment. Subsequently, fresh culture medium with appropriate reagents and/or cells was added to RIN cell cultures. Pancreatic islets were isolated from male Dark Agouti (DA) rats using the collagenase digestion method. The pancreata were minced and subsequently incubated with collagenase type V solution (1 mg/ml) in PBS at 37°C for 10 min with vigorous shaking. After incubation, HBSS was added to stop the digestion. The islets were handpicked and seeded for the experiments into 96-well flat-bottom plates (4 × 10^1^/well) in culture medium (10% FCS). The islets were used in experiments after an overnight rest. Lymph node cells (LNC) were isolated from cervical lymph nodes from DA rats and spleen cells from spleens of Albino Oxford (AO) rats, DA rats, CBA mice, Balb/C mice and C57BL/6 mice. For the purification of T cells from LNC of DA rats, anti-rat CD3-biotin conjugated antibody (BD Biosciences), MACS streptavidin microbeads and MACS separation columns were used according to the instructions of the manufacturer (Miltenyi Biotec, Aubum, CA). The obtained cells were more than 98% positive for CD4 or CD8 as deduced by cytofluorometry (FACS Calibur, BD Biosciences), and were stimulated with plate bound anti-CD3 (1 μg/ml) and anti CD28 (1 μg/ml) antibodies (eBioscience, San Diego, CA). The population of CD3^- ^cells, obtained by the same procedure as cells not bound to CD3-biotin conjugated antibody were more than 98% negative for CD3 (as deduced by cytofluorimetry) and were stimulated with LPS (1 μg/ml, Sigma). Cell free supernatants of CD3^+ ^and CD3^- ^cultures were collected after 48 hours of cultivation. RIN cells and LNC (1 × 10^6^/well) were co-cultivated in the absence or presence of tissue culture inserts (Nunc, Denmark). Spleen cell cultures (5 × 10^6^/ml) were stimulated with concanavalin A (ConA, 2.5 μg/ml) for 48 hours and subsequently cell-free supernatants (Sn) were collected. Except in experiments where 10% Sn was combined with 32 μM PA, 40% Sn was employed. α-Methyl-D-mannoside (10 mg/ml) was used to neutralize the biological activity of ConA. The animals were obtained from the breeding facility of the Institute for Biological Research "Siniša Stanković" and were kept under standardized conditions. All experiments were conducted in accordance with local and international legislations regarding the wellbeing of laboratory animals.

### Cell viability assay

In order to asses the viability of RIN cells, pancreatic islets or LNC we used the mitochondrial-dependent reduction of 3-(4,5-dimethylthiazol-2-yl)-2,5-diphenyl-tetrazolium bromide (MTT) to formazan. At the end of appropriate treatments, cell culture supernatants were removed from the plates and MTT solution (1 mg/ml) was applied. Alternatively, pancreatic islets were collected in tubes, spun down, supernatants removed and the cell pellet dissolved in the MTT solution. Finally, in co-cultivation experiments, LNC were removed from cell cultures, spun down, supernatants removed and the residue dissolved in MTT solution. MTT solution was also added to the RIN cells remaining in the plates. Incubation with MTT lasted for 30 minutes at 37°C. Dimethyl sulfoxide (DMSO) was added to the pellet of pancreatic islets, to LNC and to plated RIN cells to dissolve the formazan crystals. The absorbance was measured at 570 nm, with a correction at 690 nm, using an automated microplate reader (LKB 5060-006, LKB, Vienna, Austria).

### Measurement of NO generation

Cytofluorimetric assay was used for direct measurement of NO release with 4-amino-5-methylamino-2',7'-difluorofluorescein (DAF-FM, 1 μM), which was added to cultures 1 hour prior to the end of the treatment period. After washing in PBS, the cells were detached, resuspended in PBS and analyzed (excitation at 488 nm, emission at 510 nm) on a FACS Calibur flow cytometer (BD Biosciences).

### Apoptosis detection and caspase-3 assay

Apoptotic cells were detected using an annexinV-FITC/EtD-III staining kit (Biotium, Hayward, CA), according to the manufacturer's protocol. Briefly, after treatment, RIN cells were detached, resuspended in Annexin Binding Buffer containing AnnexinV and EtDIII, and incubated in the dark at room temperature for 15 minutes. Subsequently, samples were diluted with four volumes of Annexin Binding Buffer and analysed with FACS Calibur flow cytometer (BD Biosciences) using CellQuest Pro software (BD Biosciences). The activity of caspase-3 was determined in cultures using the Caspase-3 DEVD-R110 Fluorimetric and Colorimetric Assay Kit (Biotium, CA), according to the manufacturer's protocol. The ability of cell lysates to cleave the specific caspase-3 substrate was quantified fluorometrically using an excitation wavelength of 485 nm and an emission wavelength of 535 nm with a microplate reader (Chameleon, Hidex, Turku, Finland). The results are expressed as amount of substrate conversion (μM), deduced from a standard curve generated from known concentrations of the dye R110.

### Western blot

Whole-cell lysates were prepared in a solution containing 62.5 mM Tris-HCl (pH 6.8, 2% w/v sodium dodecyl sulfate (SDS), 10% glycerol, 50 mM dithiothreitol (DTT), 0.01% w/v bromophenol blue, 1 mM phenylmethylsulphonyl fluoride (PMSF), 1 μg/ml aprotinin, 2 mM EDTA and were electrophoresed on a 12% SDS-polyacrylamide gel. The samples were electro-transferred to polyvinylidene difluoride membranes at 5 mA/cm^2^, using semi-dry blotting system (Fastblot B43, Biorad, Munich, Germany). The blots were blocked with 5% w/v nonfat dry milk in PBS with 0.1% Tween-20 and probed with specific antibodies to p38 and phosphorylated-p38 at 1:1000 dilution (both from Cell Signaling Technology, Boston, MA), followed by incubation with secondary antibody at 1:10000 dilution (ECL donkey anti-rabbit horseradish peroxidase (HRP)-linked, GE Healthcare, Buckinghamshire, England, UK). Detection was by chemiluminescence (ECL, GE Healthcare).

### Cell-based ELISA

The expression of inducible nitric oxide synthase (iNOS) was determined in triplicate by cell-based ELISA using specific antibody (Santa Cruz Biotechnology Inc., Santa Cruz, CA, USA), according to a previously described protocol [19]. Briefly, after adequate treatment RIN cells were fixed in 4% paraformaldehyde (PFA), endogenous peroxidase activity was quenched with hydrogen-peroxide and the cells exposed to primary anti-iNOS antibody 1:200 dilution and secondary HRP-conjugated detection antibody (GE Healthcare) at 1:2000 dilution. The substrate for HRP was 3,3',5,5'-tetra-methylbenzidine and the reaction was stopped with 1 M HCl. The absorbance was measured at 450 nm and the cells were stained with crystal violet in order to correct for differences in cell numbers. The final results were obtained by division of the absorbance at 450 nm after adding the stop solution and the absorbance at 570 nm after the additional crystal violet staining (A^450^/A^570^).

### Statistical Analysis

Data are presented as the mean +/- SD of values obtained in independent experiments. Student's *t*-test was performed for analysis of the differences between means observed in the experiments.

## Results

### Cooperative effect of PA and activated immune cell products on rat pancreatic beta cell viability

**Figure 1:**
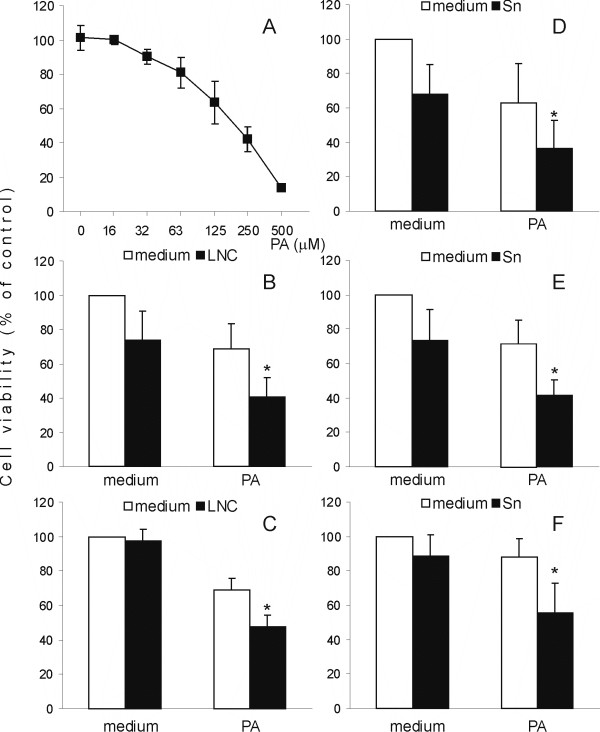
**PA and immune cells cooperate in pancreatic cell viability reduction**. RIN cells were treated with various doses of PA (A), or they were cultivated alone (medium) or co-cultivated with LNC (B, C) in the absence (B) or presence (C) of tissue culture inserts, or cultivated in the presence of 40% Sn (D) or 10% Sn (F), in the absence or presence of 125 μM PA (B-D) or 32 μM PA (F). Primary pancreatic islets were cultivated alone (medium) or in the presence of 40% Sn, in the absence or presence of 125 μM PA (E). MTT assay was performed after 20 hours of cultivation and the results are presented as the percentage of control absorbance values obtained for cultures grown in medium alone. Mean values +/- SD of values obtained in 8 (A), 14 (B), 3 (C), 11 (D), 3 (E) and 6 (F) individual experiments are presented. *p < 0.05 represents a statistically significant difference between values obtained from RIN cell cultures treated with PA in the presence of LNC and any other culture of RIN cells or RIN cells treated with PA and Sn and any other culture of RIN cells.

We co-cultivated RIN cells and rat lymph node cells (LNC) stimulated with ConA (2.5 μg/ml) in the absence or presence of PA. In preliminary experiments the PA concentration of 125 μM had a significant, yet limited effect on RIN cell viability (Fig 1A), so this dose was used in subsequent experiments. After 20 hours of treatment, either PA or activated LNC decreased RIN cell viability, but the effect was markedly greater if RIN cells were co-cultivated with activated LNC in the presence of PA (Fig 1B). Interestingly, if direct RIN-to-LNC contact was prevented with tissue culture inserts, LNC were unable to reduce RIN cell viability, but they still cooperated with PA in reducing RIN cell number (Fig 1C). This result implied that soluble mediators produced by activated immune cells cooperated with PA in inducing RIN cell death. Such an assumption was confirmed, as supernatants obtained from cultures of ConA-stimulated rat spleen cells (Sn) also efficiently cooperated with PA in RIN cell destruction (Fig 1D). In order to eliminate the possibility that the observed effects of Sn were mediated through direct action of ConA upon RIN cells, Sn were treated with α-methyl-D-mannoside (10 mg/ml) with the aim of blocking ConA activity. The blocker did not interfere with the effect of Sn on RIN cell viability (data not shown), thus excluding the possibility of direct influence of ConA on RIN cells. Importantly, Sn and PA also cooperated in the reduction of pancreatic islets isolated from DA rats (Fig 1E), thus showing that the observed effect was not specific for the transformed insulinoma cell line, but that it had relevance for beta cells in general. Finally, with the purpose of investigating if lower concentrations of PA and Sn also cooperate in reduction of RIN cell viability, RIN cells were treated with 32 μM PA and 10% Sn. As presented in Fig 1F, individually PA and Sn had just a limited inhibitory effect on RIN cells (12% and 11.4%, respectively) but their joint effect was pronounced (44.6%). This suggested that PA and Sn acted synergistically upon RIN cells.

### Sn and PA induce apoptosis of RIN cells

**Figure 2:**
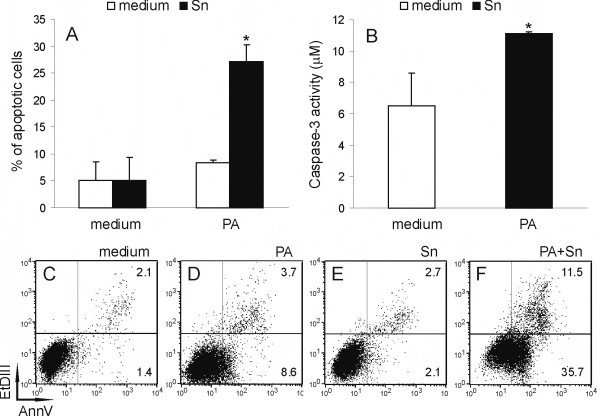
**Apoptotic cell death of RIN cells under the influence of PA and Sn**. RIN cells were cultivated in the absence (medium) or presence of 125 μM PA and/or 40% Sn for 6 hours (A, C-F) or 4 hours (B). Subsequently, the cells were stained with AnnV/EtD-III and cytofluorimetric acquisition and analysis were performed (A, C-F) or they were analyzed for caspase-3 activity (B). Mean values +/- SD of values obtained in 3 (A) and 4 (B) individual experiments with similar results are presented. *p < 0.05 represents a statistically significant difference between values obtained from cultures of RIN cells treated with PA and Sn and any other culture of RIN cells. Plots of the cells stained with AnnV/EtD-III from a representative experiment are presented in the lower panel (C-F), where numbers in the lower right quadrant of the plots are the percentage of apoptotic cells and numbers in the upper right quadrant are the percentage of necrotic cells.

Our next aim was to explore if the observed decrease in RIN cell viability was a consequence of apoptosis induction. Therefore, we analyzed AnnexinV-FITC and Et-DIII-stained RIN cells cytofluorimetrically. While PA or Sn obtained from DA rat spleen cells applied alone did not induce significant apoptosis in RIN cells after 6 hours of incubation, simultaneous application of PA and Sn induced early apoptotic changes in more than 20% of the cells (Fig 2A, C–F). Importantly, the pro-apoptotic effect was also observed after just 2 hours of treatment (5–10% of apoptotic cells), showing the rapidity of the influence. These results once again indicated that PA and Sn cooperate in a synergistic fashion. Moreover, increased activity of the pro-apoptotic cleaving enzyme caspase-3 was found in RIN cells subjected to the simultaneous treatment with PA and Sn (Fig 2B). Thus, we could conclude that concurrent application of PA and Sn decreased RIN cell viability through caspase-3 dependent induction of apoptosis.

### T cell cytokines cooperate with PA in the reduction of RIN cell viability

**Figure 3:**
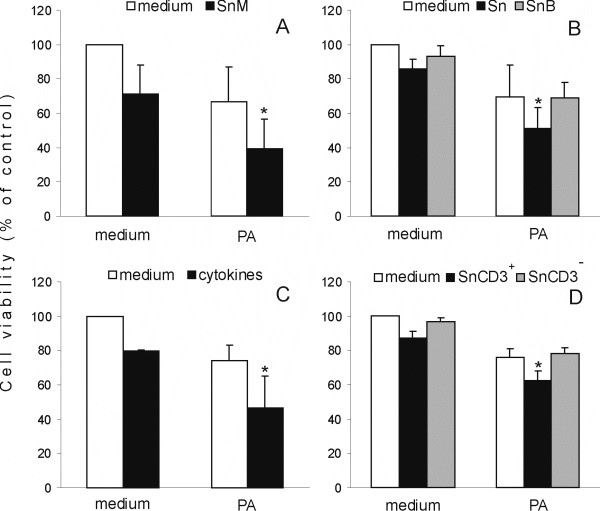
**Cooperation of mouse Sn, heated Sn, cytokines, CD3^+^Sn with PA in reduction of RIN cell viability**. RIN cells were cultivated in the absence (medium) or presence of 125 μM PA and/or 40% C57Bl/6 mouse Sn (SnM – A), and/or 40% Sn or Sn boiled for 10 minutes (SnB-B), and/or 10 ng/ml IL-1β, 10 ng/ml IL-6, 10 ng/ml IFN-γ, 10 ng/ml TNF-α, 50 ng/ml IL-17 and 100 ng/ml IL-2 (cytokines – C) and/or 40% Sn obtained from CD3^+ ^LNC or CD3^- ^LNC (SnCD3^+^, SnCD3^- ^– D). MTT assay was performed after 20 hours of cultivation and the results are presented as the percentage of control absorbance values obtained in cultures grown in medium alone. Mean values +/- SD of values obtained in 11 (A), 7 (B), 5 (C) and 4 (D) individual experiments with similar results are presented. *p < 0.05 represents a statistically significant difference between values obtained from cultures of RIN cells treated with PA and SnM (A) or PA and Sn (B) or PA and cytokines (C) or PA and SnCD3^+ ^(D) and any other culture of RIN cells.

In order to investigate the nature of the component(s) of Sn responsible for the observed cooperation with PA, Sn made from C57BL/6 mouse spleen cells were tested for efficiency in cooperation with PA. Importantly, Sn obtained from C57BL/6 mouse spleen cells were as efficient in the reduction of RIN cell viability as Sn obtained from rat cells, both individually and in cooperation with PA (Fig 3A). Moreover, similar efficiency was observed for Sn obtained from CBA and Balb/C mice or AO rats (data not shown). Thus, we could conclude that the soluble factor(s) involved in the cooperation with PA were not species or strain-specific. Furthermore, we tested the resistance of the soluble component(s) to heat by boiling Sn for 10 min before use in the experiments. Exposure of Sn to heat led to loss of the cooperative cytotoxic action of Sn and PA (Fig 3B) indicating that heat-sensitive components were responsible for the cooperation with PA in RIN cell death induction. The possibility that cytokines present in the Sn were involved in this cooperation was investigated by treating RIN cells with the combination of IL-1β, IFN-γ, TNF-α, IL-17, IL-2 and IL-6 in the absence or presence of PA. This combination of cytokines alone reduced RIN cell number and also cooperated with PA in the reduction of RIN cell viability (Fig 3C). The effect was similar to that of Sn, so we could conclude that cytokines might be responsible for the cooperative reduction of RIN cell viability by Sn and PA. Finally, since Sn were products of mixed populations of cells, including T cells, macrophages, B cells and other cell types, LNC were separated into CD3^+ ^cells (T cells) and CD3^- ^cells (all the other cells), by magnetic bead purification, and the obtained populations were used for production of Sn, in order to determine their relative contribution to the observed cooperation. Importantly, Sn obtained from CD3^+ ^cells stimulated with anti-CD3 and anti-CD28 antibodies cooperated with PA in the reduction of RIN cell viability (Fig 3D). On the other hand, Sn obtained from CD3^- ^cells stimulated with LPS did not cooperate with PA in RIN cell destruction. Hence, we could conclude that T cells were producers of the cytokines responsible for the cooperation with PA.

### PA+Sn-induced RIN cell death is dependent on p38 MAPK activation

**Figure 4:**
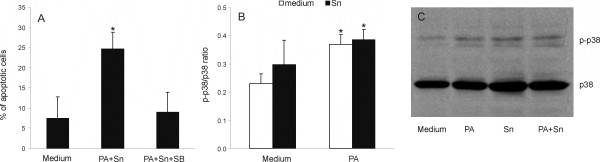
**The involvement of p38 MAPK signaling pathway in the induction of apoptosis of RIN cells under the influence of PA and Sn**. RIN cells were cultivated in the absence (medium) or presence of 125 μM PA and/or 40% Sn. For apoptosis detection, RIN cells were treated with PA and Sn in the absence or presence of an inhibitor of p38 MAPK signaling – SB202109 (40 μM) for 6 hours, and subsequently stained with AnnV/EtD-III (A). Alternatively, RIN cells were treated with PA and/or Sn for 1 hour, then cell lysates were made before western blotting for p38 MAPK and phosphorylated p38 (p-p38) (B, C). Mean values +/- SD of values obtained in 5 (A) and 3 (B) individual experiments with similar results are presented, or a representative western blot (C). *p < 0.05 represents a statistically significant difference relative to the cultures grown in medium without additional treatment (A, B) and to the cultures treated with SB (A).

Our further goal was to determine if p38 MAPK-signaling is involved in PA+Sn-induced apoptosis in RIN cells. As the first step, SB202109, a selective p38 MAPK inhibitor, was applied to RIN cultures treated with the combination of PA and Sn, and this inhibitor was shown to prevent PA+Sn-triggered apoptosis in RIN cells (Fig 4A). Accordingly, PA+Sn-induced p38 MAPK activation was detected in RIN cells (Fig 4B), thus supporting the importance of p38 induction for PA+Sn induced apoptosis in RIN cells. However, PA, but not Sn, was able to activate p38 to the levels observed in cultures treated with both PA and Sn. Thus, it seems that p38 activation is not enough for efficient initiation of RIN cell apoptosis, but that it is necessary for induction of the death of these cells by coordinate action of PA and Sn.

### Hemoglobin protects RIN cells from PA+Sn-induced apoptosis

**Figure 5:**
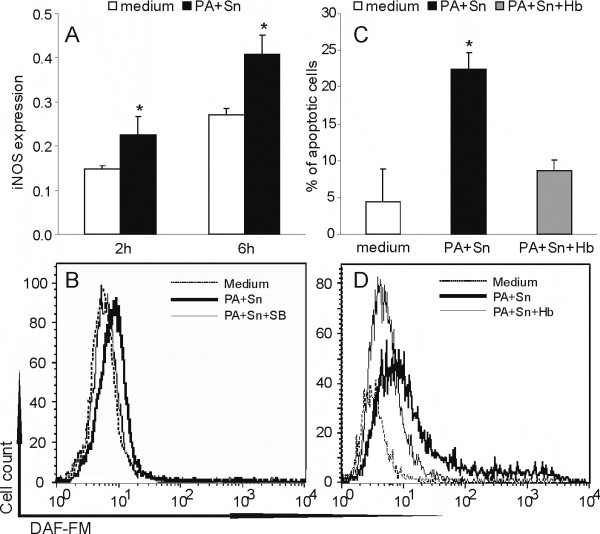
**PA and Sn induce RIN cell apoptosis through induction of nitric oxide production**. RIN cells were cultivated without treatment (medium) or treated with Sn and/or PA and/or hemoglobin (Hb, 20 mg/ml) and/or SB202109 (40 μM). After 2 or 6 hours RIN cells were fixed before cell-based ELISA for iNOS (A). Cells were stained with DAF-FM (B, D) and AnnV/EtD-III (C) after 6 hours of cultivation. Mean values +/- SD of values obtained in three individual experiments with similar results are presented (A, C). Alternatively, plots from a representative of at least three experiments with similar data are presented (B, D). *p < 0.05 represents a statistically significant difference relative to the cultures grown in medium without additional treatment (A, C) and to the cultures treated with Hb (C).

We also explored if the effect of PA and Sn on RIN cell viability was indirect, through induction of nitric oxide (NO) production in these cells. Inducible nitric oxide synthase (iNOS) expression measured by cell-based ELISA showed that PA+Sn induced expression of this protein in RIN cells, after 2 and 6 hours of incubation (Fig 5A). Additionally, the treatment led to NO production in RIN cells, while hemoglobin, a NO quencher, down-regulated the levels of free NO (Fig 5B) and accordingly protected RIN cells from apoptotic cell death induced by PA and Sn (Fig 5C). Importantly, the p38 signaling inhibitor – SB202109 that protected RIN cells from the cytotoxicity also inhibited NO release in RIN cells (Fig 5D). Thus, these results clearly suggest that NO produced in RIN cells in a p38-dependent manner contributes to PA+Sn-induced RIN cell apoptosis in a major way.

## Discussion

In the present paper we show that cytokines produced by T cells cooperate with a saturated free fatty acid in the induction of beta cell apoptosis. The observed cooperation is synergistic both at the level of the intensity and the speed of the action. Importantly, the cooperative effect was also observed if primary pancreatic islets were used as the target population, thus excluding the possibility that the cytotoxicity was specific for the transformed cell line.

There is an increasing number of reports indicating the importance of saturated fatty acids or soluble products of immune cells, including cytokines and NO in the reduction of beta cell mass during diabetes pathogenesis [8,11,18]. Nevertheless this is the first report to our knowledge, that describes cooperation between a saturated fatty acid and soluble immune mediators in the destruction of pancreatic beta cells. The fact that soluble mediators cooperate with PA is clearly shown in the experiments where RIN cells and LNC were separated with tissue-culture inserts, and substantiated with the corresponding results obtained with cell-free Sn. Moreover, similar efficiency of Sn from various strains of rats and mice, clearly suggests that product(s) responsible for the observed cooperation are not species- or strain-specific. Furthermore, the inability of heat-treated Sn to cooperate with PA in RIN cell destruction implies that the soluble product(s) are heat-sensitive, i.e. proteins. The obvious protein candidates are cytokines, as IL-1β, IL-6, IFN-γ and TNF-α have been shown to be efficient in β-cell destruction [8,11,17,18]. Additionally, we previously reported that IL-17 contributes to NO-dependent cytotoxicity of beta cells [20]. Moreover, one of the major constituents of Sn – IL-2 was found to cooperate with PA in the activation of Jak-3 and STAT-5 in human lymphocytes [21]. Therefore, we used the combination of IL-1β, IL-6, IFN-γ, TNF-α, IL-17 and IL-2 and were able to mimic the effects of Sn on RIN cells. Finally, the observed ability of Sn obtained from pure CD3^+^, but not CD3^- ^cells, to cooperate with PA in reducing RIN cell viability indicates that T cells are the major cell population responsible for the production of the soluble product(s) which cooperate with PA in the process. Importantly, the cytokines we used to mimic the effect of Sn are either typical T-cell products (IL-2, IFN-γ, IL-17) or they can be produced by various cells including T cells (IL-6, IL-1β, TNF-α). This is in agreement with the observed ability of pure CD3^+ ^cell culture supernatants to affect RIN cell viability. Also, if we take into account that some cytokines (IL-6, IL-1β, TNF-α) can be produced by B cells, macrophages and other CD3^- ^cells, and that production of all of the cytokines by T cells could be supported by CD3^- ^cells, it becomes clear why CD3^+ ^cells produced a "weaker" supernatant than lymph node cells. Moreover the inability of CD3^- ^cells to provide a supernatant effective against RIN cells, leaves us with T lymphocytes as the prime suspects for the cooperation with fatty acids in the destruction of beta cells in diabetes.

Furthermore, we can conclude that the major signaling pathway responsible for the induction of apoptosis in RIN cells under the coordinated influence of PA and Sn is the p38 MAPK pathway. This conclusion is based on the p38-activation observed under the influence of PA and Sn, as well as on the complete protection of RIN cells from the apoptosis induction by the specific p38 signaling inhibitor. It is well known that p38 is involved in induction of apoptosis [22], and more specifically in the induction of apoptosis of beta cells, under the influence of both cytokines [23,24] and PA [25]. In our hands PA, but not Sn, was able to activate p38 in RIN cells. However, although PA potently induced p38 in RIN cells, it did not cause massive apoptosis. Importantly, with the same level of p38 activation under the cooperative action of PA and Sn, a substantial proportion of RIN cells underwent apoptotic changes, while inhibition of the p38 signaling pathway prevented the induction of apoptosis in RIN cells. Thus, it seems that activation of p38 *per se *is not enough for apoptosis induction in RIN cells, but at the same time p38 activation is probably necessary for the observed cooperative induction of apoptosis in these cells.

Finally, the major effector molecule responsible for RIN cell apoptosis may be NO. Namely, the NO scavenger Hb protects RIN cells from the deleterious effect of PA and Sn, while the p38 signaling inhibitor, which inhibits PA+Sn-imposed apoptosis of RIN cells, also prevents PA+Sn-induced NO synthesis in these cells. The importance of NO for induction of beta cell apoptosis by cytokines has been repeatedly reported, while the role of NO in fatty acid induced death of beta cells is still controversial. For instance, it was shown that IL-1 or TNF-α induced cell death in INS-1 cells and that IL-1, but not palmitate or oleate potently induced iNOS gene expression and NO generation in these cells [8]. There are, however, findings indicating the ability of primary beta cells to produce NO and express both constitutive and inducible isoforms of NOS under the influence of PA [25-27]. In our experiments, massive production of NO was observed in RIN cells under the coordinated action of PA and Sn. The novelty of our finding is that PA could contribute to NO induction in the inflammatory milieu, represented in our experiments by Sn. Generally, it is thought that NO-dependent destruction of beta cells is characteristic of T1D, where immune cells infiltrate pancreatic islets and produce NO-inducing cytokines, while on the contrary fatty acids do not provoke NO-dependent cell death in T2D [28]. Here, we show that PA is able to cooperate with Sn in inducing NO synthesis. Thus, it seems reasonable to assume that free fatty acid levels could be very important for the destruction of beta cells during ongoing inflammation in the pancreas. Such a reasoning favors hypotheses that inflammation is important for the development of T2D, as well as that obesity is a predisposing factor for T1D [4,5].

Taken together, as it was shown that both NO and p-38 signaling contribute to apoptosis induction in RIN cells under the influence of PA and Sn in a major way, it is tempting to speculate that in our system the cooperative effect of PA and Sn is dependent on activation of p38, which in turn leads to generation of NO and destruction of RIN cells. Once NO is generated, p38 might play an important role in the predisposition of RIN cells to NO-imposed apoptosis, as genetic down-regulation of p38α was previously shown to lower the sensitivity of beta cells to cell death induced by a NO donor [24]. Importantly, besides its role in classical induction of apoptosis, p38 activity has been assigned a major role in endoplasmatic reticulum (ER) stress-induced apoptosis [29]. Also, NO produced in beta cells under the influence of cytokines has been shown to contribute largely to the induction of ER-stress in beta cells [30]. Thus, the possibility of the involvement of the ER stress components in the observed effect of PA and cytokines on beta cells should be investigated in the future.

## Conclusion

Our results imply that limited hyperlipidemia and inflammation, when acting in concert, may carry out a powerful attack upon pancreatic beta cells. The cooperative cytotoxic effect of palmitate and soluble T cell products described herein has clear significance for understanding the pathogenesis of diabetes, as it suggests that there might be direct cooperation between factors of obesity and inflammation in beta cell destruction. However, as our data are from a simplified *in vitro *experimental system, it requires future *in vivo/ex vivo *studies to provide insights about the feasibility of the observed cooperation in living organisms.

## Abbreviations

ConA: concanavalin A; DA: Dark Agouti; DAF-FM: 4-amino-5-methylamino-2',7'-difluorofluorescein; FCS: fetal calf serum; Hb: hemoglobin; HRP: horse radish peroxidase; IFN: interferon; IL: interleukin; iNOS: inducible nitric oxide synthase; LNC: lymph node cells; MAPK: mitogen-activated protein kinase; MTT: 3-(4,5-Dimethylthiazol-2-yl)-2,5-diphenyl-tetrazolium bromide; NO: nitric oxide; PA: palmitic acid; PBS: phosphate buffered saline; Sn: cell-free supernatants of spleen cell cultures treated with ConA for 48 hours; T1D: type 1 diabetes; T2D: type 2 diabetes; TNF: tumor necrosis factor.

## Authors' contributions

TC carried out most of the experimental procedures and helped to draft the manuscript. IS and SS-G participated in the design and coordination of the study and helped to draft the manuscript. GT performed Western blot analysis. DjM conceived the study, organized and coordinated it and conducted some of the experimental procedures. All authors read and approved the final manuscript.
